# Supplementary material for: Estimation of DNA Degradation in Archaeological Human Remains
Source: Genes (Basel). 2023 Jun 9;14(6):1238. doi: 10.3390/genes14061238 (PMC10298407; doi:10.3390/genes14061238)
Supplement: Supplementary file 1 [file genes-14-01238-s001.zip › Figure S1.pdf]

ESTIMATION OF DNA DEGRADATION IN ARCHAEOLOGICAL HUMAN REMAINS

Antonella Bonfigli[1,#], Patrizia Cesare[1,#], Anna Rita Volpe[1], Sabrina Colafarina[1], Alfonso Forgione[2], Massimo Aloisi[1], Osvaldo Zarivi[1,§,*], and Anna Maria Giuseppina Poma[1,§]

Figure S1: Electrophoretic analysis of sonicated DNA samples

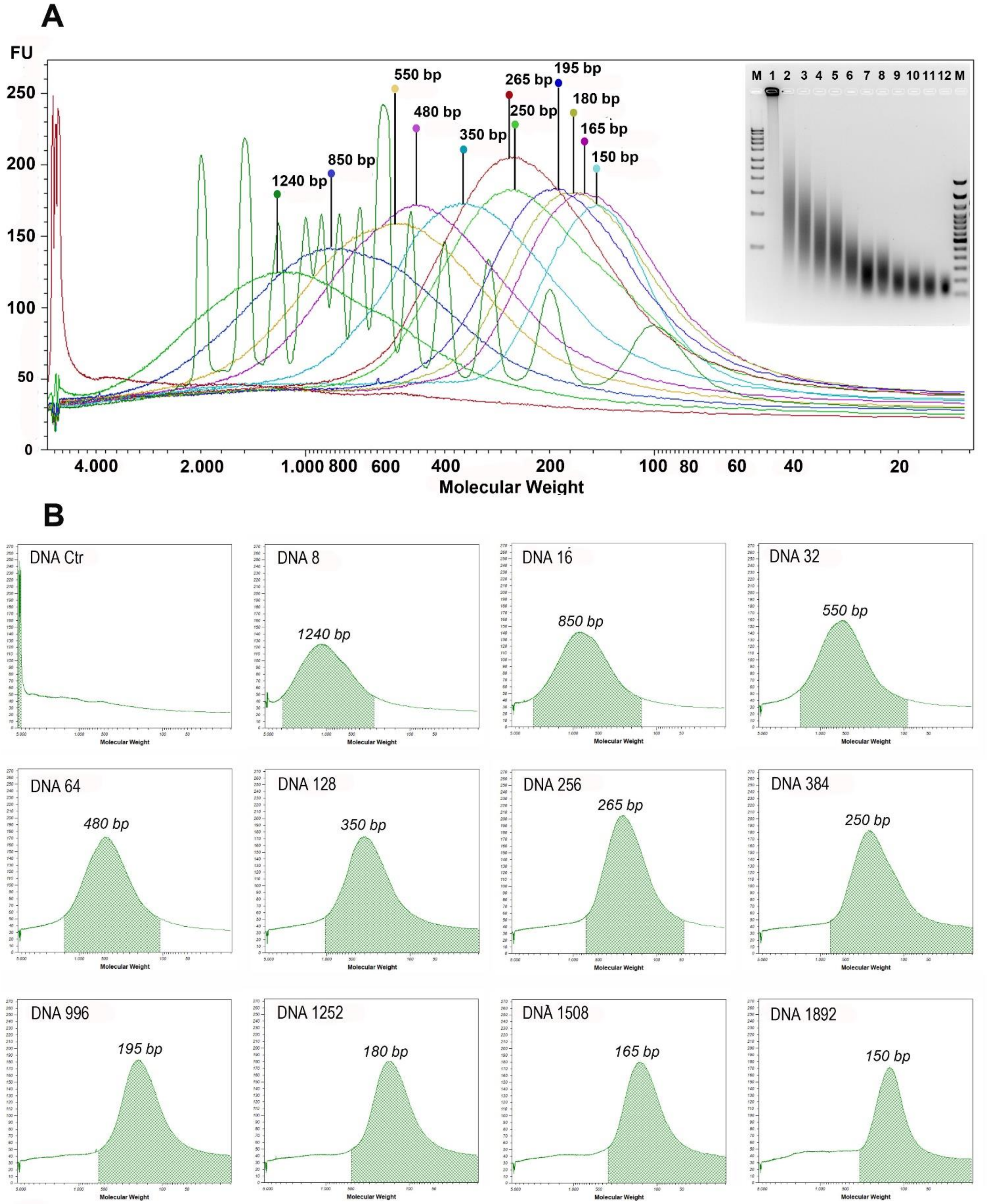

Figure S1- Electrophoretic analysis of sonicated DNA samples. Fragmentation of standard DNA obtained with the Vibra-Cell™ Ultrasonic sonicator MODEL VCX 400 (Sonix) at various sonication times. A: electrophoretic run of the samples in 1.4% agarose gel, lane 1 1kb DNA ladder Sigma Aldrich, lane 2 control DNA, lanes 3-13 sonicated DNA at increasing times, lane 14 10bp DNA ladder Invitrogen and gel analysis with the TotalLab 1D v14.1 software with the distribution of the fragments produced in relation to the dimensions; B: distribution of fragments and the mean size in base pairs for the various sonication times.
