# Supplementary material for: Estimation of DNA Degradation in Archaeological Human Remains
Source: Genes (Basel). 2023 Jun 9;14(6):1238. doi: 10.3390/genes14061238 (PMC10298407; doi:10.3390/genes14061238)
Supplement: Supplementary file 1 [file genes-14-01238-s001.zip › Figure S2.pdf]

ESTIMATION OF DNA DEGRADATION IN ARCHAEOLOGICAL HUMAN REMAINS

Antonella Bonfigli[1,#], Patrizia Cesare[1,#], Anna Rita Volpe[1], Sabrina Colafarina[1], Alfonso Forgione[2], Massimo Aloisi[1], Osvaldo Zarivi[1,§,*], and Anna Maria Giuseppina Poma[1,§]

Figure S2 ALU_50 Standard curve Ct versus pg/µl

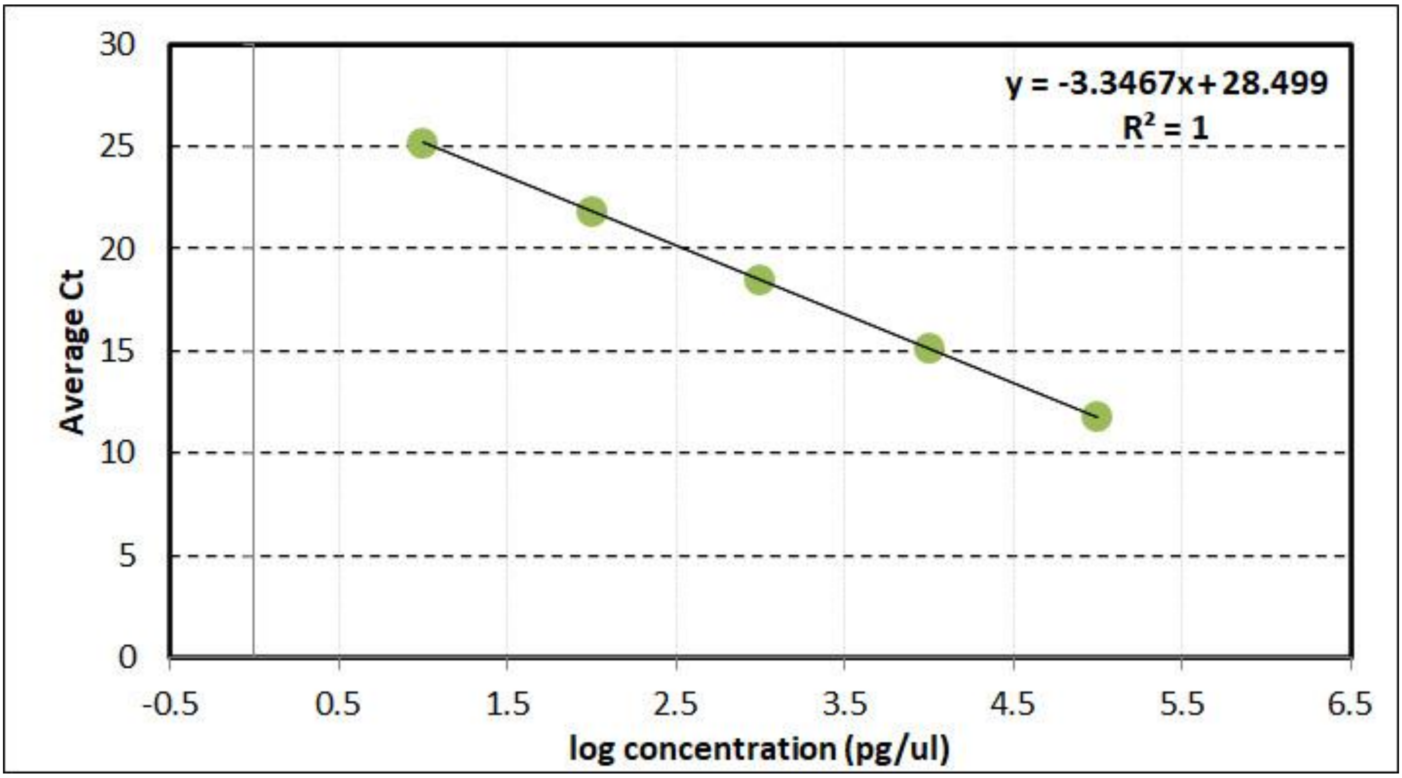

Figure S2 – ALU_50 Standard curve Ct versus pg/µl. The standard curve was obtained by qPCR amplification of 1 µl of standard DNA (SD1111 Thermo Scientific) from 100,000 pg/µl to 10 pg/µl, with 1:10 serial dilutions, with the following primer pair: F_ALU_50/R_ALU_50. The Ct are a function of the log of the concentration expressed as pg/µl.
