# Supplementary material for: Estimation of DNA Degradation in Archaeological Human Remains
Source: Genes (Basel). 2023 Jun 9;14(6):1238. doi: 10.3390/genes14061238 (PMC10298407; doi:10.3390/genes14061238)
Supplement: Supplementary file 1 [file genes-14-01238-s001.zip › Figure S3.pdf]

ESTIMATION OF DNA DEGRADATION IN ARCHAEOLOGICAL HUMAN REMAINS

Antonella Bonfigli[1,#], Patrizia Cesare[1,#], Anna Rita Volpe[1], Sabrina Colafarina[1], Alfonso Forgione[2], Massimo Aloisi[1], Osvaldo Zarivi[1,§,*], and Anna Maria Giuseppina Poma[1,§]

Figure S3: Standard curves, Ct versus copy number/µl, for the 18S rRNA gene

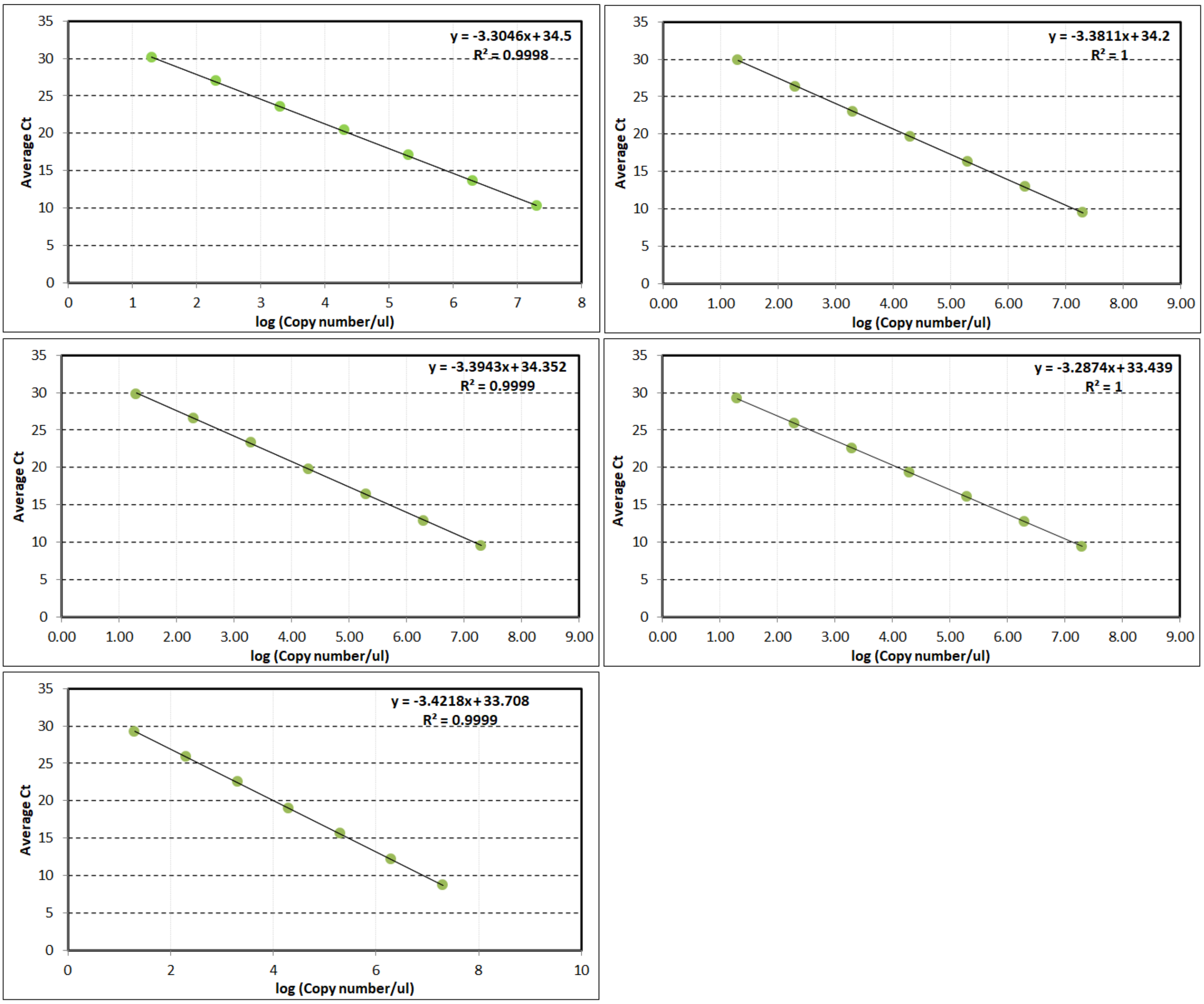

Figure S3 | Standard curves, Ct versus copy number/µl, for the 18S rRNA gene. The standard curves were obtained by qPCR amplification of 1 µl of the 802 bp fragment, containing the 18S rRNA gene, from 20,000,000 to 20 copies/µl, with 1:10 serial dilutions, with a single forward primer F_18S and the following reverse primers: R_18S-61 (A), R_18S-118 (B), R_18S-158 (C), R_18S-253 (D), R_18S-332 (E), with these pairs of primers amplicons of increasing size are obtained. The Ct are a function of the log of the concentration expressed as copy number/µl.
