# Supplementary material for: Estimation of DNA Degradation in Archaeological Human Remains
Source: Genes (Basel). 2023 Jun 9;14(6):1238. doi: 10.3390/genes14061238 (PMC10298407; doi:10.3390/genes14061238)
Supplement: Supplementary file 1 [file genes-14-01238-s001.zip › Figure S4.pdf]

ESTIMATION OF DNA DEGRADATION IN ARCHAEOLOGICAL HUMAN REMAINS

Antonella Bonfigli[1,#], Patrizia Cesare[1,#], Anna Rita Volpe[1], Sabrina Colafarina[1], Alfonso Forgione[2], Massimo Aloisi[1], Osvaldo Zarivi[1,§,*], and Anna Maria Giuseppina Poma[1,§]

Figure S4: Standard curves Ct versus log copy number/µl, for the 12S rRNA gene.

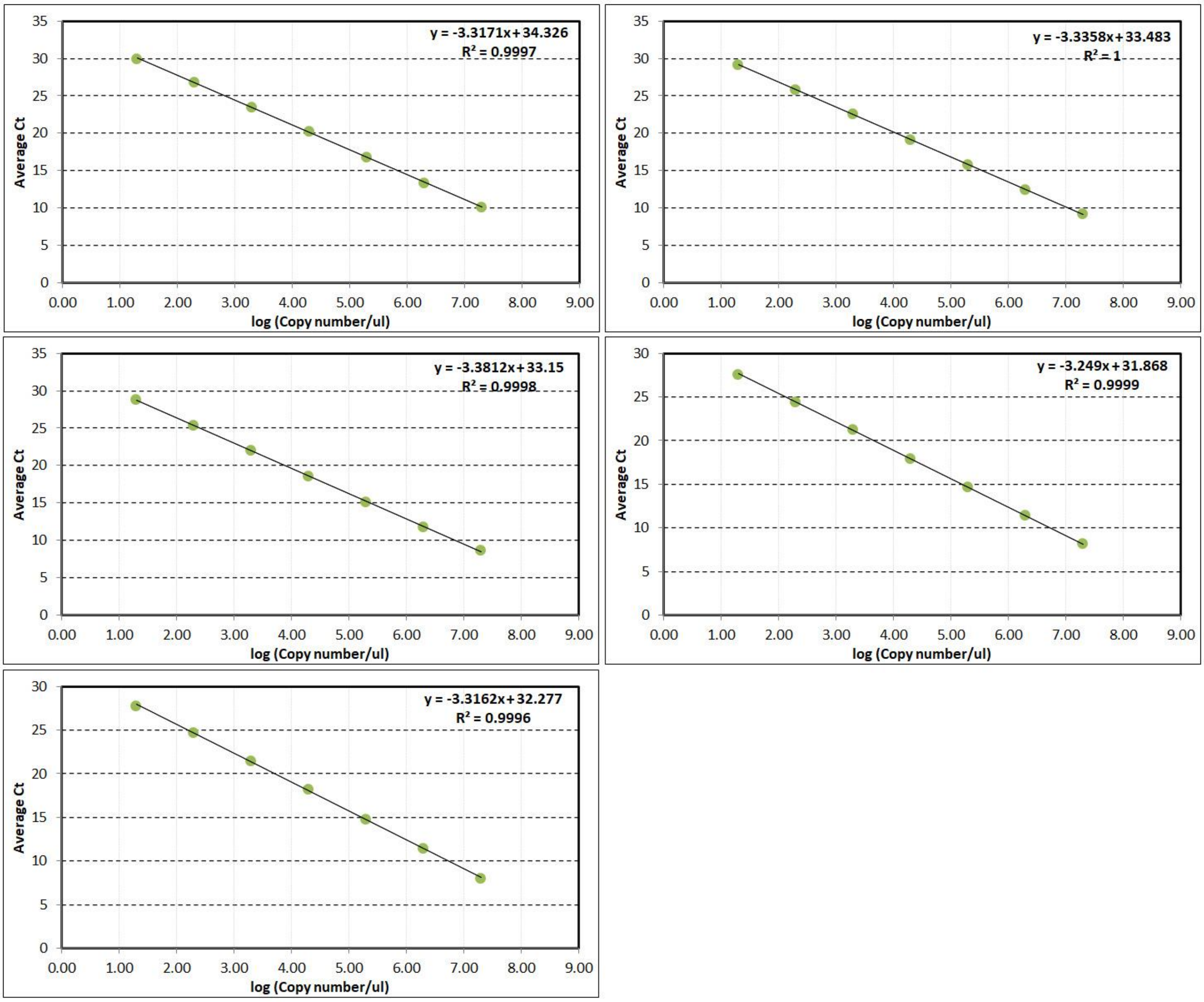

Figure S4- Standard curves Ct versus log copy number/µl, for the 12S rRNA gene. The standard curves were obtained by qPCR amplification of 1 µl of the 2,119 bp fragment, containing the 12S rRNA gene, from 20,000,000 to 20 copies/µl, with 1:10 serial dilutions, with a single forward primer F_12S and the following reverse primers: R_12S_59 (A), R_12S_95 (B), R_12S_152 (C), R_12S_219 (D), R_12S_281 (E), with these pairs of primers amplicons of increasing size are obtained. The Ct are a function of the log of the concentration expressed as copy number/µl.
